# Leaf and Stem-Based Dew Detection Algorithm *via* Multi-Convolutional Edge Detection Networks

**DOI:** 10.3389/fpls.2022.861534

**Published:** 2022-04-25

**Authors:** Meibo Lv, Pengyao Zhou, Tong Yu, Wuwei Wang, Daming Zhou

**Affiliations:** School of Astronautics, Northwestern Polytechnical University, Xi’an, China

**Keywords:** dew from leaves and stems, dew detection, dew measurement, mathematical morphology, edge detection (ED)

## Abstract

During the process of drought and rehydration, dew can promote the rapid activation of photosynthetic activity and delay the wilting time of plant leaves and stems. It is clear that the amount of dew will affect the growth of plants. However, limited research is being done to detect and measure the amount of dew. Therefore, in this study, a statistical method for measuring the amount of dew based on computer vision processing was developed. In our framework, dewdrops can be accurately measured by isolating the background area based on color features and detecting the edge and statistical area. In this scheme, the multi-convolutional edge detection networks based on contour search loss function are proposed as the main implementation algorithm of edge detection. Through color feature background region segmentation and the proposed edge detection networks, our algorithm can detect dew in complex plant backgrounds. Experimental results showed that the proposed method gains a favorable detection accuracy compared with other edge detection methods. Moreover, we achieved the best Optimal Image Scale (OIS) and Optimal Dataset Scale (ODS) when testing with different pixel values, which illustrate the robustness of our method in dew detection.

## Introduction

Dew attaches to the leaves and stems of plants and allows photosynthesis during the hydration process, delaying the retention of plant leaves in dry conditions. As a result, how to detect the dew area to calculate dew amount becomes one of the effective means to present plant growth status. With the development of computer image processing technology, it is possible to use images to calculate dewfall. Since dewdrops falling on the leaves tend to be irregular and small in size, it is usually employed with dewdrops of size 1–5 mm diameter. It is difficult to estimate the error of the statistical dewdrop area caused by noise or the uncertainty of the background environment. The detection of these dewdrops often depends on soil and environmental moisture, since there are few algorithms present to calculate the area of dewdrops.

A portable weighing microanalyzer, which uses micro-pressure elements to automatically record the amount of dewdrops, is used (Heusinkveld et al., 2006). This method requires large-scale installation and observation of all plants in a certain area, but it is not possible to detect every dewdrop. For instance, Jacobs et al. (2006) express the methods of the surface energy budget dew model algorithm and the eddy current covariance technology algorithm to calculate the amount of dewdrops. However, first, the surface energy budget model cannot perform year-round dew detection due to frost conditions or snow cover; second, the eddy current covariance technique underestimates the nighttime fluxes under low wind and very dewy conditions. All-weather dewdrop detection cannot be achieved by these two methods. Jiftah et al. (2010) describe a newly developed photosynthetic and transpiration rate detector for detecting the response of transpiration and photosynthesis to dewdrop on leaves. However, this detection method mainly relies on manual detection and dew transpiration to measure the amount of dew, which exposes the shortcomings of the detection algorithm, that is, poor timeliness and lack of ability to accurately measure the amount of dewdrops.

By reviewing the dewdrop detection method mentioned earlier, we adopted a method of background region segmentation based on color features. First, the sharp color areas in the image were removed to reduce the interference of background color on dewdrop detection. Second, to obtain the dewdrop contour and reduce the error of dewdrop area detection, we conducted edge detection on crop dewdrop images with low background noise.

In this study, we proposed a dewdrop detection method based on mathematical morphology. At first, the image color features are extracted for background processing of crops, where the mathematical morphology algorithm is used to reduce the influence of outside lights and other disturbances on dewdrop measurement. Then, the edge detection algorithm is used to process the dewdrop region to obtain the contour shape of dewdrops. Finally, the contour features of dewdrops are extracted and the information of dewdrop size and density is calculated. In edge detection, we compared the traditional (Canny, 1986; Ziou and Tabbone, 1998; Liu et al., 2021) and Laplace (Marr and Hildreth, 1980; Wang and Yang, 2009) algorithms with the holistically nested edge detection (HED) (Xie and Tu, 2015), crisp edge detection (CED) (Wang et al., 2017), and richer convolutional features for edge detection (RCF) (Liu et al., 2017) algorithms based on Deep Learning (Wu et al., 2019). We found that when Canny operator detected a picture with 816,000 pixels, the detection time was 13.4 ms, while Laplace operator detected the same picture in 13.8 ms, but their detection accuracy was only 0.557 and 0.446. Canny algorithm needs to detect the edge by manually selecting the threshold. In this study, the best combination of threshold is (64,81). HED (Xie and Tu, 2015)/RCF (Liu et al., 2017) algorithms detected the same picture for 3,029/2,698 ms, and their detection rates were 0.833 and 0.876. After weighing the advantages and disadvantages of the above algorithms, a multi-convolutional edge detection network based on the loss function of threshold hyperparameter based on contour search is proposed. The novel contributions of this study are summarized as follows:

• Contour search loss function based on contour search is defined in training multi-scale neural network to fully consider the problem of noise aggravation caused by the increase of convolution layers in dew detection. And the area surrounded by dew is used as the threshold to suppress the interference of noise area.
• We used a new idea of HSV segmentation before detection. The sensitivity of HSV segmentation of color is used to suppress the recognition of complex background noise by a multi-scale neural network to reduce the influence of strong plant color interference on dew detection.
• In the detection algorithm, we have created multi-convolutional edge detection networks suitable for detecting dewdrop edges, which can be helpful to extract the edges of dewdrops.

## Related Work

In this section, we review the traditional detection methods. Furthermore, edge detection methods of dew are then discussed. Finally, works based on edge detection are presented.

### Detection Scheme

There are few studies on measuring the amount of dew on crops by images, most of which are based on sensors to detect dew intensity on crops. For example, dew was collected by absorbent metal and carbon foam based on large gaps (Kotzen, 2015) and dew collection based on water condenser (Tomaszkiewicz et al., 2015). Photonic crystal fiber interferometer in the reflection model (Mathew et al., 2011) and flat drosometer (Barradas and Glez-Medellín, 1999) are commonly used to observe the onset and the amount of dew. Despite the fact that these sensors and collection devices are fast, they cannot collect the amount of dew on the whole blade nor reflect the actual dew data. For observing crop growth conditions, it is impossible to do accurate observation. Meanwhile, any contact surface placed near the ground can become contaminated quickly with dirt and may require frequent maintenance during long-term testing. A method of using blotting to assess the amount of dew on vegetation (Richards, 2004), in which measurements are made by pressing a pre-weighed blotting-paper sheet onto a wet leaf and then weighing the leaf, is used. Although this method is accurate, it is cumbersome and requires manual intervention, which can be difficult to implement during the detection of dewdrops on crops in large areas. A temperature sensor (Yan et al., 2022) covered by a humidity-sensitive thin film (referred to as HSTF) is proposed. The measurement mechanism is that the mutation of the temperature-time curve caused by the thermal effect of the HSTF can be used to detect dew points. Although the detection speed of this method is fast because its detection depends on the influence of temperature, the change of ambient temperature will bring systematic errors to the detection results. Combining color and thermal imaging to detect the free water on the surface of dew and other things (Ghalyani and Mazinan, 2019) can well distinguish dew from plants. However, its dependence on illumination conditions was a drawback. Thermal imaging proved to be useful when using high-resolution cameras for water droplet detection but was dependent on ambient weather conditions.

Therefore, many researchers put their thoughts on computer vision to detect dew. At the same time, a Canny-based algorithm to detect the amount of dew condensed on glass (Zhu et al., 2014) is proposed. For reducing lighting errors, the glass is chosen to be transparent when covered with water and opaque when dry and is used as a carrier for dew condensation. However, this detection method is susceptible to the complex background due to the permeability of glass. In addition, if the method is applied, there will be a need for manual adjustment parameters. Similar to this detection method, another detection method using the Sobel edge detection operator (Zhou et al., 2013) has been developed, but the experiment shows that this detection method is suitable for the scene where the difference between water droplets and background is obvious, and it is not introduced for the environment where the difference is not obvious. At the same time, an algorithm (Liao et al., 2013) including histogram equalization, frame intersection, binarization, and shape checking has been developed to detect water drops. However, this algorithm has the same weakness as the Sobel edge detection operator. In addition, an algorithm (Cord and Gimonet, 2014) that segments the image and then uses either watershed or background subtraction to identify water drops by using size and shape to identify real raindrops is proposed. However, by adding more frames, this algorithm not only improves performance but also adds delay to the overall system operation time. In addition, a method of detecting leaf wetness by fusing color and thermal imaging technologies (Swarup et al., 2021) has been developed, but this idea could not resist the influence of external color and light interference.

### Edge Detection

In the field of edge detection, there are plenty of algorithms to use. The Canny algorithm proposed by Canny first denoises the input image and obtains pixels of possible edges by calculating the image gradient. The Laplace algorithm determines the position of the edge by applying the zero crossing between the quadratic differential property and the peak value. Through the analysis of these two most representative traditional edge detection principles, it was found that they have the characteristics of fast detection speed and good real time. However, it cannot filter the background noise well nor smooth the image and requires manual intervention. Therefore, the edge detection method of Deep Learning (Wu et al., 2019) is applied to detect the edge of dewdrops. In edge detection, the HED (Xie and Tu, 2015), the RCF (Liu et al., 2017), and the CED (Wang et al., 2017) algorithms are the most representative ones. The CED (Wang et al., 2017) algorithm was based on the HED (Xie and Tu, 2015) algorithm through the post-optimization path. They used an efficient sub-pixel convolution stepwise upward sampling function to generate edge effect images that were better aligned with image boundaries. Similar to the HED (Xie and Tu, 2015) algorithm, CED (Wang et al., 2017) has the edge sharpening and poor edge acquisition capability of small and weak targets (Xie and Tu, 2015). All of the algorithms obtain image edges by superimposing features of the convolution layer, while the difference is that the HED (Xie and Tu, 2015) and CED (Wang et al., 2017) algorithms are stacked by the features of the last layer of each convolution layer, while the RCF (Liu et al., 2017) algorithm is stacked by the features of each convolution layer to form edges, whereas, for the detection of dewdrop edge, the recognition rate of noise can be improved by using the features of each layer because the dewdrop contour is small. Despite the fact that the detection accuracy of the RCF (Liu et al., 2017) algorithm is not enough to detect most dewdrop contours, the multi-convolutional edge detection networks proposed here can effectively solve this problem.

Thus, a dewdrop detection scheme in accordance with background region segmentation, which is based on color features and edge detection proposed in this study, can effectively improve the shortcomings of the above algorithms.

**FIGURE 1:**
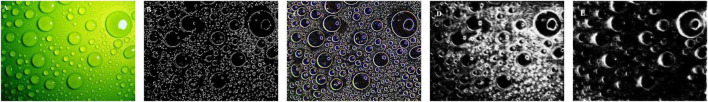
Four algorithms are applied on a leaf for dewdrop detection. **(A)** Image, **(B)** Canny, **(C)** Laplace, **(D)** HED (Xie and Tu, 2015), **(E)** RCF (Wang et al., 2017).

Through the investigation of the above algorithms (Figure 1), it was found that there were various defects in the traditional algorithm and Deep Learning (Wu et al., 2019) algorithm for measuring dew amount on the leaf area of crops. For the traditional algorithms represented by Canny and Laplace, they have the defects of low suppression of background noise and great influence of human intervention. For Deep Learning (Wu et al., 2019) algorithms represented by HED (Xie and Tu, 2015), RCF (Liu et al., 2017) and CED (Wang et al., 2017), they all get the edge of the image by superimposing the features of convolution layer, but for dew detection, because the outline of dew is very small, improving the ability of dew recognition will also increase the interference of background noise. In order to make up for the defects of the four edge detection algorithms, an improved edge detection method is proposed with the assistance of contour search as the threshold value and the loss function, as well as dewdrop area statistics based on the background region segmentation of color features. Through this improved algorithm, those four algorithms can be effectively eliminated. For the dew edge, the dew amount on the window can be accurately measured, making it possible to accurately observe the linear effect of dew amount on plant photosynthesis, delay the wilting time of plant leaves, and reflect plant growth activity linearly.

## Materials and Methods

### Algorithm Summary

At present, the existing dew amount observation devices at home and abroad can be mainly divided into two types: mechanical type and optical detection type. The principle of mechanical dew gauge is to calculate the amount of dew contained in the inner container at a certain time, to evaluate the dew amount level and the amount of precipitation; the scattering or refraction of light by dewdrops in the light field is used in the optical detection dew gauge to measure the size of dewdrops in the dew field; the mechanical dew gauge is more accurate in detecting the amount of dew, but cannot meet the needs of real-time detection; the optical detection type can meet the real-time requirements. Most of them are only used to estimate dew amount by the dewdrop size and cannot obtain the accurate dew amount.

In view of the limitations of the two existing methods above, the dewdrop image analysis method based on image processing presents great potential for development. On the one hand, designing suitable image processing algorithms will achieve accurate measurement of a single image; on the other hand, increasing the speed of the algorithm operation for long periods of time makes the observation of dew amount possible to combine both accuracy and real-time performance.

In this study, we designed an algorithm to implement the following image processing functions, taking the color disturbance background image of the crops during dew condensation as an example:

• We used the method of background region segmentation based on color features to segment the target detection image to remove the influence of external color noise on dew detection.
• The edge detection of the region-segmented image is performed, and the contour shape of dewdrops is obtained by optimizing the detection results through mathematical morphology.
• We use the Irregularly Raindrop and Dewdrop Detection (IRRD) algorithm to detect the dew area and obtain all dew areas in the whole map by superposition.

**FIGURE 2:**
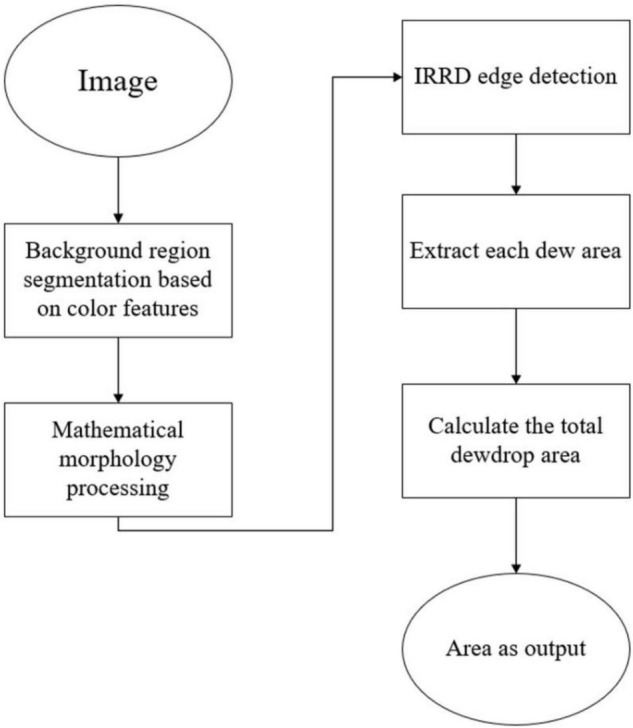
Algorithm flowchart. We can effectively solve the influence of complex background color noise on dew detection by detecting the amount of dew after HSV segmentation and edge detection. In the process of edge detection, the Irregularly Raindrop and Dewdrop Detection (IRRD) algorithm is innovatively proposed, making our detection more powerful for noise suppression.

The flowchart of the algorithm is given in Figure 2:

There is no data set specially used for the detection of dewdrop edges. We have designed and utilized a dewdrop simulation system to collect dewdrop photographs and make a dewdrop data set in accordance with the format of the HED-BSBD_PASCAL data set.

**FIGURE 3:**
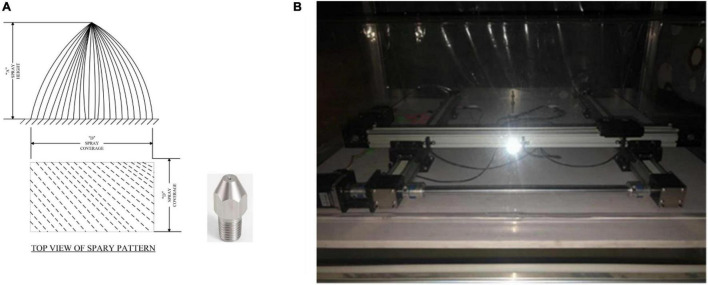
The **(A)** device for water droplets simulation, where the nozzle is used to simulate the situation of dewdrops when it falls. Meanwhile, the intensity of dew is simulated by water pressure. The **(B)** device for window simulation. Through the 3-DOF slide, the incline of the window can be simulated, while the camera is not in the vertical line of the window. The simulated leaves dewdrop images obtained in this way can reduce the error of angle detection. We used the water drop simulation system to simulate the formation of dew and take photographs of dew at the time and season of dew generation.

By studying the physical properties of water droplets, we have found that dew and water droplets have similar edge profiles. To obtain the pictures of water droplets, we designed a water droplets simulation system, where water droplets are simulated to form dew (Figure 3).

### Background Region Segmentation Based on Color Features

In common cases, the environment of the crops is very complex, which results in background interference such as reflections of light on the image of the crops in addition to dewdrops. For reducing the impact of the window scene on the dewdrop analysis algorithm, pre-processing is needed for the complex background. The common out-of-window scenes are abundant in color information and can be analyzed with color features. The color-rich regions are segmented out and will not be analyzed for dewdrops to avoid the influence of complex background on feature extraction algorithms such as edge detection.

Among color images, RGB images are the most common color format and serve as the color standard in the industry. The HSV model is a color space based on the intuitive characteristics of color, where H is the hue that determines the basic color of a region and S is the color saturation that measures how close the color is to the spectral color. The larger S is, the closer the color will be to the spectral color, and the less white light will be mixed in. V characterizes luminance in visual perception.

**FIGURE 4:**
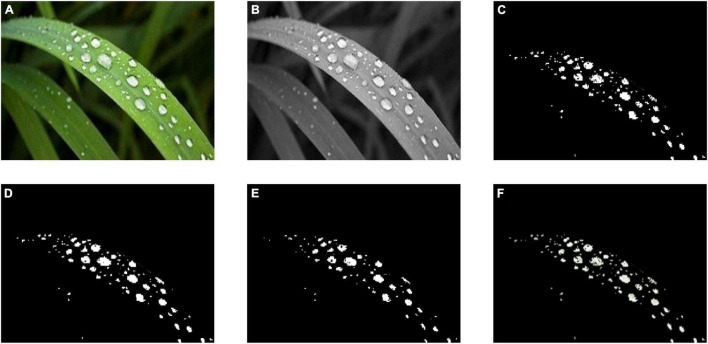
Segmentation results of HSV. **(A)** is the dew image, **(B)** expresses the saturation component of HSV color space, **(C)** shows the result of HSV saturation segmentation, **(D)** reflects the result of mathematical morphology closing operation, **(E)** is the result of mathematical morphology corrosion operation, and **(F)** is the dew image after HSV segmentation. Because dew refracts the ambient color, through HSV segmentation and mathematical morphology processing, we can well filter the interference of environmental color on dew detection.

The process of image segmentation with the HSV color space is shown in Figure 4, where Figure 4A is the crop’s image with dewdrops and Figure 4B is the saturation component of the HSV color space. It can be seen in the figure that there is an obvious light interference at the bottom right, and there is a red area above which will affect the subsequent algorithms such as edge detection. At first, the original image was converted to HSV color space as described above.


$$I_{H\,S\,V}=H\,S\,V\,\left(I_{R\,G\,B}\right)$$

A threshold was then set to remove the oversaturated areas with the color saturation component S:


$$I_{C}=I_{S}<\alpha$$

Where *I*_*C*_ is the area removed by color saturation component S, and *I*_*S*_ is S saturation component area. α is the threshold value, and the result obtained in this case, α = 50, was selected as shown in Figure 4C. The noise area *I*_*C*_ was eliminated by •E which is a mathematical morphological closure operation:


$$I_{C}=I_{C}•E$$

where E is the morphological structure element and • is the closed operator definition operator. In this example, the structure element was chosen as a square with a side length of 25, and the obtained result is indicated in Figure 4D. To better overcome the influence of the region boundary, the area of the region was reduced with the application of the mathematical morphological erosion operation. The structure element was selected as a square with a side length of 25, and the result obtained is shown in Figure 4E. The corresponding partitioned area is shown in Figure 4F.

After the background region segmentation based on color features, we filtered out the bright color area of the image taken from the window to provide a good input image for the gray-scale image of the edge detection.

### Multi-Convolutional Edge Detection Networks for Dewdrop Detection (Irregularly Raindrop and Dewdrop Detection)

Inspired by the RCF (Liu et al., 2017) algorithm, we proposed an Irregularly Raindrop and Dewdrop Detection (IRRD) neural network detection algorithm for dewdrop detection. ResNet101 (He et al., 2016; Wang et al., 2021) network is used as the backbone to fuse multi-layer convolutional features of different depths, making itself detect dewdrop edge more accurately.

#### Data Set

**FIGURE 5:**
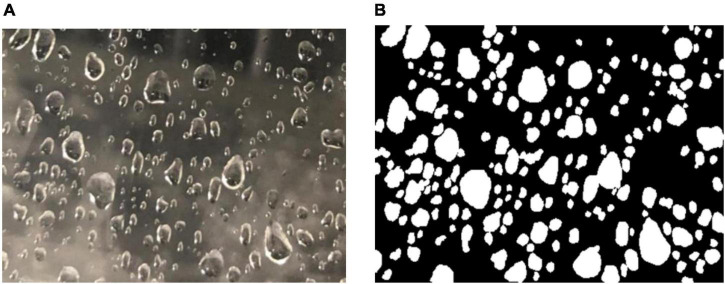
Panel **(A)** is the water drop picture collected by simulation equipment, and panel **(B)** is the picture of **(A)** marked by Labelme software.

Figure 3A generates water droplets to simulate dew and the 3-DOF of Figure 3B is used to simulate the angle change of shooting dew in the real scene. The water drop photographs collected by this simulation system can effectively simulate the dew in the real scene. Therefore, high object variation was ensured. Images were collected by mobile phone, with a camera resolution of 1706 × 1279 and an aperture of F2.8. The 3-DOF workbench was used to adjust the shooting angle so that the images are of different scales. Figure 5 shows an example image. We randomly selected five time points in the morning and evening to take a total of 372 dew photos. In this way, the weather conditions such as sunshine and solar terms of dew can be better simulated. We randomly selected 300 photographs from these 372 photographs and generated 512 photographs through data enhancement methods of cropping, rotation, and scaling. These images are marked with open-source software called Labelme. After that, we randomly select 100 pictures as the testing set and made the remaining 412 pictures as the training set. It is worth noting that we can better supplement the influence of incomplete dew edge information collection caused by insufficient camera field of view on edge recognition by cutting and rotating. Scaling is used to provide rich scale changes for data sets.

#### Data Training

**FIGURE 6:**
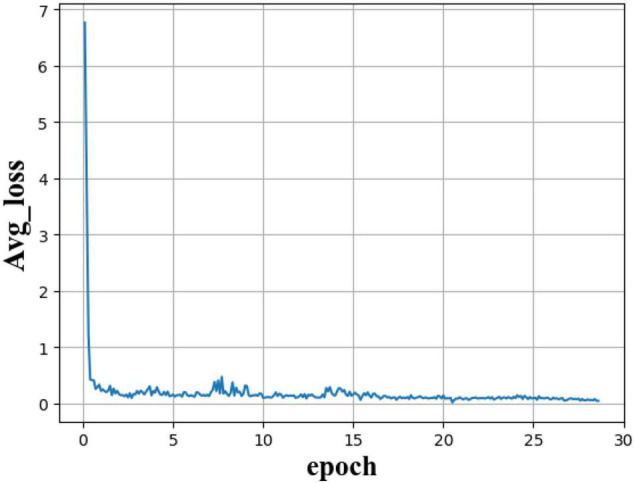
A training loss plot of our model. Through the change of average loss value, we can see that with the deepening of training, the loss value tends to be around 0.04 and remains stable. It can be seen that the data set of 412 pictures can make our model converge.

All frameworks were trained on an end-to-end basis in a single RTX2070 GPU at the initial learning rate of 0.01 and the epoch of 30. In our model, we used the ReLU activation function to initialize the parameters. The networks were trained 12,360 times with the X9TIR notebook, and we kept the weights according to the training every 1,000 times. The average loss value was used to see the convergence characteristics of training (as shown in Figure 6). Finally, the loss value stabilized at around 0.04.

#### Structure of Multi-Convolutional Edge Detection Network

**FIGURE 7:**
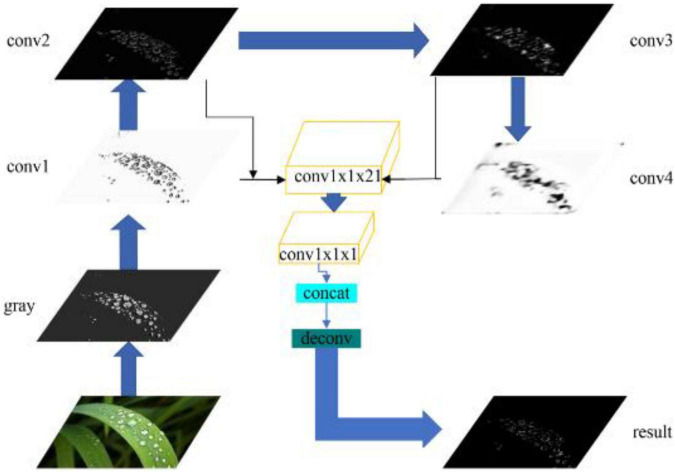
The architecture of the multi-convolutional edge detection network.

Compared with VGG16 (Simonyan and Zisserman, 2015; Qin et al., 2021), ResNet101 (He et al., 2016; Wang et al., 2021) deepens the number of network layers, possesses the residual function to solve the problem that gradient dispersion causes in the network, to fail to converge, and the accuracy decreases. Referring to the idea of RCF (Liu et al., 2017) algorithm for the VGG16 (Simonyan and Zisserman, 2015; Qin et al., 2021) network improvement, we improved the ResNet (He et al., 2016; Wang et al., 2021) network. The multi-convolutional edge detection network structure is shown in Figure 7:

We added conv1x1x21, conv1x1x1, and loss/RelU by eliciting each convolutional layer. The results of the four convolution layers were superimposed in the same dimension using conv1x1x21, conv1x1x1, and Concat to form the final result.

**FIGURE 8:**
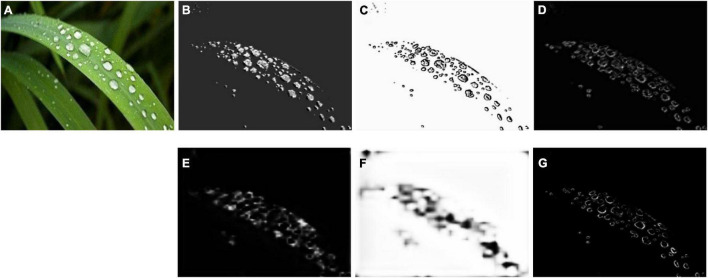
Effect of each layer of multi-convolutional edge detection networks. **(A)** Original image, **(B)** ground truth, **(C)** gray-level image, and **(D–G)** effect of four convolutional layers.

The multi-convolutional edge detection network is used as the backbone of the RCF (Liu et al., 2017) edge detection algorithm for dewdrop particle detection, of which the effect of each layer is shown in Figure 8.

To better feel the impact of these convolution layers on the detection accuracy, we conducted the study by removing these five convolution layers, respectively. The results are shown in Table 1.

**TABLE 1:** Influence of different convolution layers on detection accuracy.

Conv layers	IRRD[Ap(%)]
Conv1.2.3.4.5	85.56
Conv1.2.3.4.final_out	85.45
Conv1.2.3.5.final_out	84.27
Conv1.3.4.5.final_out	84.47
Conv2.3.4.5.final_out	85.06
Conv1.2.3.4.5.final_out	88.44

We can see that the detection accuracy of 816,000 pixels is between 0.8506 and 0.8556, when only the features of the five-layer convolution network are used. When we use a multi-scale fused neural network to detect the edges by using all the features in the network, our detection accuracy is improved to 0.8844. Compared with the 1- to 5-layer fusion feature of detection accuracy, our detection accuracy is improved by 0.0288. It can be seen that the accuracy of edge detection will be improved when all convolution features are used.

**FIGURE 9:**
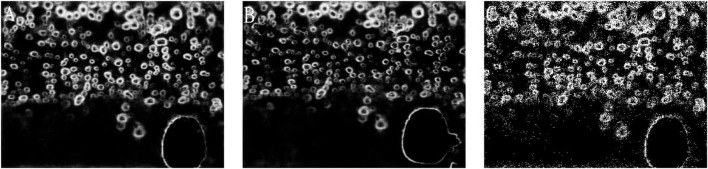
Panel **(A–C)** images are the weight detection results generated when the batch_size is 1.2.4 in turn.

When the size of batch_size is changed, the effect of multi-convolutional edge detection networks will be different. Due to the limited computing power of RTX2070 GPU, we tested the same picture by using the weights obtained by different batch_size of 1, 2, and 4 to show the influence of batch_size on the detection results of the IRRD algorithm (Figure 9). By comparing the detection results of batch_size of 1, 2, and 4, we find that the detection accuracy for the multi-convolutional edge detection networks is the best when batch_size is 1.

#### Loss Function Based on Edge Lookup as the Threshold

Since the data set is usually labeled by multiple markers, despite the difference in individual perception, the results of various people have a high degree of consistency. For each image, we used the average markers of each person to generate a probability map for the presence of edges. For each point, 0 means that no marker thinks that the point is an edge, and 1 means that every marker thinks that the point is an edge. A hyperparameter η is defined here: If the probability of a point being an edge is greater than η, then this point will be considered as an edge; if the probability of this point is 0, then it will not be an edge; furthermore, points that are considered to have a probability between 0 and η will be considered as controversial points and will not be counted in the loss function. Therefore, the loss function of each point can be written as follows:


$$l\,\left(X_{i};W\right)=\left\{ \begin{array}{c} \alpha •\log\left(1-P\,\left(X_{i};W\right)\right)\;\,i\,f\,\,y_{i}=0\\ 0\;i\,f\,\,0<y_{i}<\eta \\ \beta •\log P\,\left(X_{i};W\right)\;i\,f\,\,o\,t\,h\,e\,r\,w\,i\,s\,e \end{array}\right.$$

where,


$$\alpha =\lambda •\frac{\frac{\left|Y_{\left|S^{+}\right|}^{+}\right|}{\left|Y_{\left|S^{+}\right|}^{+}\right|+\left|Y_{\left|S^{-}\right|}^{+}\right|}}{\left|Y^{+}\right|+\left|Y^{-}\right|}•\frac{\left|S^{+}\right|}{\left|S^{+}\right|+\left|S^{-}\right|}$$

$$\beta =\frac{\frac{\left|Y_{\left|S^{+}\right|}^{+}\right|}{\left|Y_{\left|S^{+}\right|}^{+}\right|+\left|Y_{\left|S^{-}\right|}^{+}\right|}}{\left|Y^{+}\right|+\left|Y^{-}\right|}•\frac{\left|S^{+}\right|}{\left|S^{+}\right|+\left|S^{-}\right|}$$

where |*Y*^+^| denotes the number of points that must be edges on the way, |*Y*^−^| denotes the number of points that must not be edges on the way, |*S*^+^| indicates the area with the largest detection. |*S*^−^| represents the total area of dewdrop area, λ is hyperparameterized, the edge of the largest pair is $\left|Y_{\left|S^{+}\right|}^{+}\right|$, the corresponding edge point for the other regions is $\left|Y_{\left|S^{-}\right|}^{+}\right|$ the corresponding edge point for the other regions is, and |$\bar{\text{S}}$| is the average of the other areas.

The feature vector at pixel i and the result of whether it is an edge are represented as*X*_*i*_ and *Y*, respectively, P(X) is a standard sigmoid activation function, and W represents all the learning parameters in the network. Therefore, the loss function of the whole picture can be written as follows:


$$L\,\left(W\right)=\sum_{i=1}^{\left|I\right|}\left(\sum_{k=1}^{K}l\,\left(X_{i}^{\left(k\right)};W\right)+l\,\left(X_{i}^{f\,u\,s\,e};W\right)\right)$$

where $X_{i}^{\left(k\right)}$ denotes the feature vector of stage k, $X_{i}^{f\,u\,s\,e}$ denotes the feature vector of stage fusion, |*I*| represents the number of pixels, and K represents the number of stages.

Through the function set in this way, the segmentation edge interference caused by HSV segmentation can be eliminated and the real dewdrop area on the window can be identified. Area input will be realized for the later implementation of dewdrop area statistics.

## Results

### Dewdrop Contour Extraction

After the edge detection result was obtained, the dewdrop particles were visible in small sizes. Therefore, it is possible for the edges of the area segmented by HSV to be observed during the detection. At this stage, the detection result is applied by open operation filtering at first, then hole filling, and finally by designing a filter to filter out the segmented area with larger area to get the dewdrop map.

**FIGURE 10:**
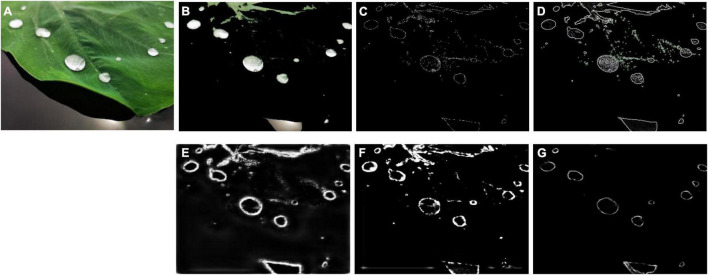
Edge detection results of five algorithms. **(A)** Original image, **(B)** the result of HSV, **(C)** the result of Canny, **(D)** the result of Laplace, **(E)** the result of HED (Xie and Tu, 2015), **(F)** the result of RCF (Wang et al., 2017), and **(G)** the result of IRRD.

Compared with traditional methods such as indirect dew measurement using micro-pressure elements, the IRRD algorithm uses vision to directly measure dew amount. It has a positive guiding significance for reducing systematic errors. Therefore, we use the edge-based dew detection method. Compared with the other four algorithms that cannot form closed edges and cannot detect all the edges of dewdrops, the IRRD algorithm based on searching edge loss function can effectively detect the closed edges of dewdrops (Figure 10).

In addition, the evaluation indexes of edge detection of these five algorithms are shown in Table 2.

**TABLE 2:** Five algorithm performance indicators.

	Canny	Laplace	HED (Xie and Tu, 2015)	RCF (Wang et al., 2017)	IRRD
Ap of edge (%)	55.7	44.6	83.3	87.6	88.4
Ap of dew area (%)	12.4	6.7	27.3	34.4	96.2
ODS	0.61	/	0.783	0.804	0.811
OIS	0.63	/	0.805	0.825	0.871
Computation time(s)	0.0134	0.138	3.029	2.698	2.753
Data size	521(b)	1.61(kb)	2.74(kb)	2.85(kb)	2.53(kb)

Among them, Ap of an edge is the index to evaluate the detected edge, and Ap of dew area is the index to evaluate whether the edge can form a closed edge. The formula of Ap of the dew area is as follows:


$$\mathrm{Ap}\,\mathrm{of}\,\mathrm{dew}\,\mathrm{area}=\frac{\sum \left(1-\frac{S_{M\,A\,N}-S_{A\,L\,M}}{S_{M\,A\,N}}\right)}{N},$$

where *S*_*MAN*_ denotes the dew area marked by Labelme, *S*_*ALM*_ denotes the dew area after the detection algorithm, and N denotes the number of dewdrops.

**FIGURE 11:**
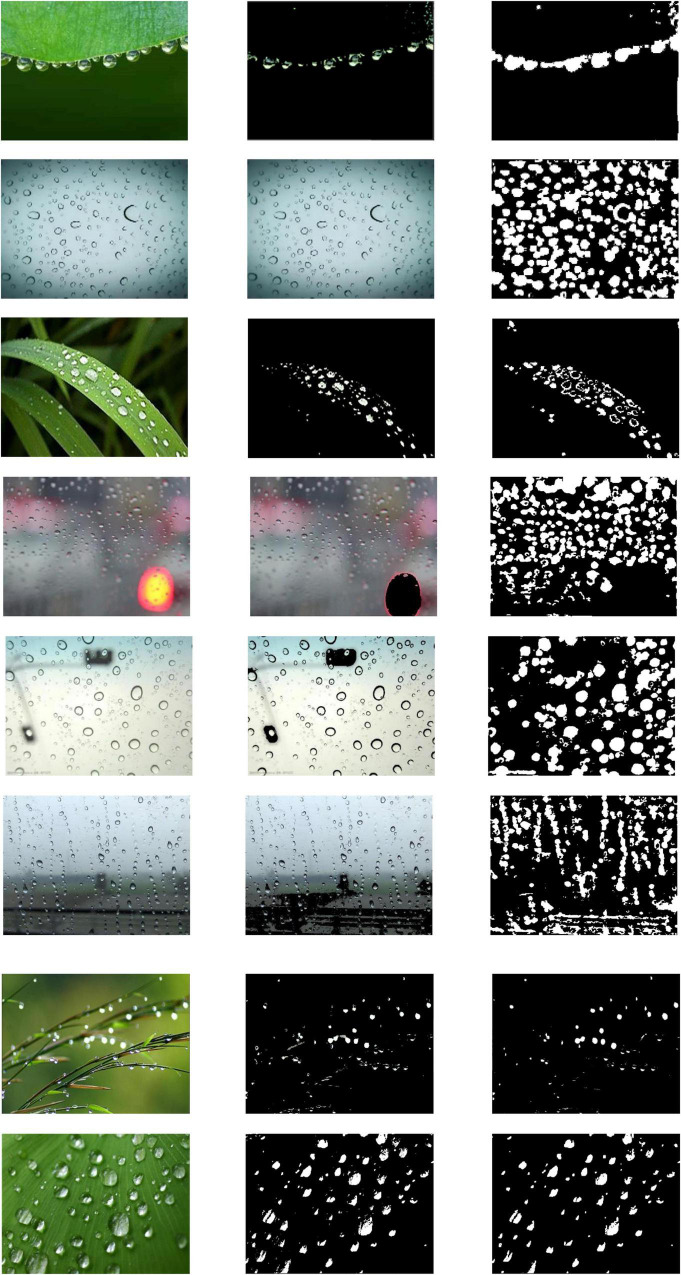
Dew area detection under three different backgrounds.

In Figure 11, we extracted the dew edge for these scenes. As a supplement to the interference of non-plant background and strong background color, we used three photographs of dew on glass sheets as supplementary photographs for detection. In the first column, the first image demonstrates a leaf with dewdrops in light background interference. The second image displays a glass with dewdrops without background interference. The third image shows leaves with dewdrops against a complex background. The fourth to sixth attached images show water droplets under strong background interference. The second column is the background region segmentation based on color features in three cases. The third column is the image detected by the IRRD algorithm.

According to Figures 10, 11, we can see that when there is external background interference, the other four algorithms can detect many false edges. Although they can detect dew edges, the highest accuracy rate for dew edges is 0.876, but it brings great interference to the subsequent detection of the dew area. The best ratio of dewdrop edge to all edges is only 0.344. When the IRRD algorithm is used, the accuracy rate reaches 0.884 and the ratio of tangent edge to all edges reaches 0.962.

### Dewdrop Area Analysis

**FIGURE 12:**
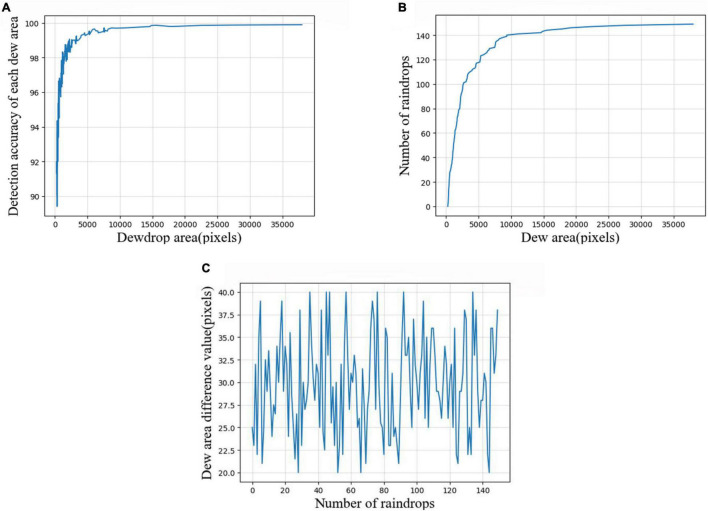
Dewdrop area analysis. **(A)** Accuracy of area detection for each dew area, **(B)** area value of each dew in the original dew map, and **(C)** the difference of each dew area between the IRRD algorithm and original image detection results.

After the obtainment of the dewdrop contour area, the shape, size, and other characteristics of dewdrops can be analyzed in a more detailed way. The specific analysis results are shown in Figure 12.

Figure 12A expresses the detection accuracy of each dew area, wherein the abscissa expresses the dew area with a pixel value as a unit, and the ordinate indicates the detection accuracy. Figure 12B shows the area value of each dew in the original picture, in which the abscissa expresses the dew area and the ordinate expresses the order of the dew area. Figure 12C shows the accuracy of the IRRD algorithm in dew area detection compared with the original image. Among them, the abscissa shows the dew area, and the ordinate shows the error of the dew area arranged by size when detected by the IRRD algorithm. When we compared Figures 12A,B, we found that the detection accuracy of dew with an area larger than 5,000 pixels by our algorithm was generally above 0.98. However, the detection accuracy of dew with an area value below 50,00 was low. For small areas, the accuracy of our detection algorithm was about 0.85. This is because the loss function of the IRRD algorithm converges with the dew area as the threshold. Other algorithms lack this key information. Moreover, the difference of the dew area is below 40 pixels.

In the next step, we calculate the histograms of all distances.

As indicated by the above analysis, the accuracy rate of our algorithm is 88.4%, which is 0.8% higher than that of other algorithms. At the same time, the ratio of dewdrop edge to all edges is 96.2%, which is 61.8% higher than that of the past algorithms. The underlying reason is that, compared with our method, the loss function of others lacks the statistics of dewdrop area and filter based on dewdrop area, thus weakening the function of detecting real dew edges. Moreover, the number of layers of multi-scale networks used by other methods is less than that of the IRRD algorithm, which inevitably leads to insufficient detection accuracy of dew edge by other methods. As a result, the ability of other methods to detect the true edge of dew is insufficient. Besides, the IRRD algorithm has more layer depth than other algorithms. Therefore, the IRRD algorithm is superior to other algorithms in detecting the dew edge.

## Discussion

### Performance Analysis

In addition, the evaluation indexes of edge detection of these five algorithms are shown in Table 2. It can be observed that the IRRD algorithm is 2.74 s longer than the traditional algorithm represented by Canny, but the accuracy is improved by 0.334. Compared with the HED (Xie and Tu, 2015) algorithm, the IRRD algorithm takes 0.276 s less and improves the accuracy by 0.051. Compared with the RCF (Liu et al., 2017) algorithm, the IRRD algorithm is 0.055 s longer, but the accuracy is improved by 0.008. When we use this contour search loss function, we can effectively improve the global convergence threshold of dew detection and the optimal threshold of a single image. The RCF (Liu et al., 2017) algorithm with the best ODS/OIS among the four algorithms increased from 0.804/0.825 to 0.811/0.871. In this study, using the background region segmentation based on color feature and IRRD algorithm to detect dew can avoid the interference of color areas and the influence of the area error of closed areas formed after color segmentation. Therefore, the IRRD algorithm has better detection results and convergence. In addition, it has the smallest data size among the three Deep Learning (Wu et al., 2019) algorithms when the weight size is consistent.

### Failure Cases

**FIGURE 13:**
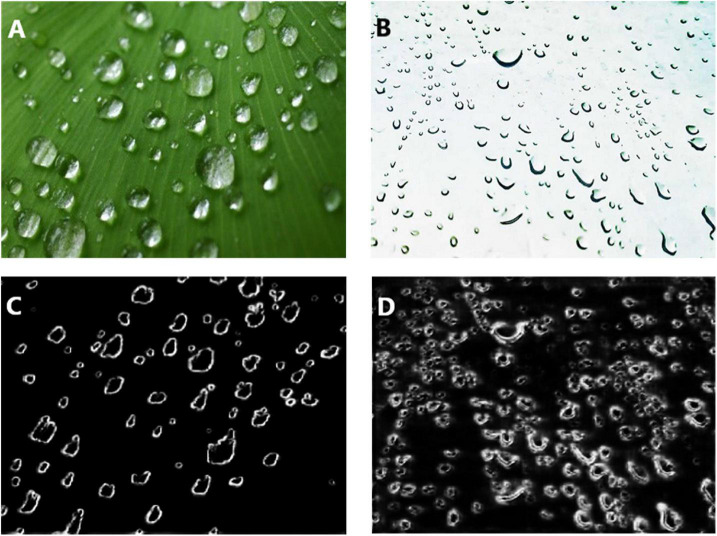
Failure cases [**(A)** the dew when the background white light is strong and **(B)** the edge of dew is the same as the background color], where **(C)** the edge of dewdrops with different thicknesses when the light is enough, and **(D)** dewdrops with incomplete edges when the color of the background is mapped to dewdrops.

We also have revealed two failure cases of the IRRD algorithm in Figure 13. Specifically, the first column shows that IRRD detects the edge of dewdrops with different thicknesses when the light is enough. We conjecture that this may be due to the IRRD training different network layers with the same supervision, which results in unfavorable feature representation in some cases. The second column of Figure 13 shows that IRRD fails to form the edge completely when the color of the background is mapped to dewdrops. It is mainly due to the HSV segmentation, which filters out the part with similar color between dew edge and background as background.

### Potential Application and Outlooks

In addition, the IRRD algorithm can also be used to detect the amount of rainfalls on the windshield and determine the actual location of raindrops. For detecting the amount of rainfall on the windshield, a good mathematical relationship can be established between the accurate rainfall detected by IRRD and the wiper speed, so that the wiper can intelligently and independently change the rotation speed according to the result of IRRD’s detection. This research is helpful to driving safety. When removing the interference of raindrops, the first step is to determine the location of raindrops. the IRRD algorithm can provide more accurate edge information of raindrops and then provide more accurate edge location information for subsequent steps.

## Conclusion

When we used this multi-scale convolutional neural networks based on contour search loss function, we solved the problem of dew detection when the background interference is complex and the color interference is strong and provided accurate input for the rapid activation of dew and photosynthetic activity and the establishment of a proper functional relationship between the withering time of plant leaves. Although the detection time of IRRD was 55 ms longer than that of RCF (Liu et al., 2017), the running memory was 0.32 kb less and the detection accuracy improved by 0.008. However, due to the high network depth in this study, the real-time performance for detecting a 1080 P image was low. In the future, network pruning and the influence of the output layer on results could be added to improve real-time detection.

## Data Availability Statement

The raw data supporting the conclusions of this article will be made available by the authors, without undue reservation.

## Author Contributions

ML and PZ designed the research and wrote the manuscript. ML, PZ, WW, and DZ conducted and analyzed the experiments. WW helped to edit the manuscript. DZ supervised the project and helped to design the study. All authors contributed to the article and approved the submitted version.

## Conflict of Interest

The authors declare that the research was conducted in the absence of any commercial or financial relationships that could be construed as a potential conflict of interest.

## Publisher’s Note

All claims expressed in this article are solely those of the authors and do not necessarily represent those of their affiliated organizations, or those of the publisher, the editors and the reviewers. Any product that may be evaluated in this article, or claim that may be made by its manufacturer, is not guaranteed or endorsed by the publisher.
